# LncRNA UCA1 promotes development of gastric cancer via the miR-145/MYO6 axis

**DOI:** 10.1186/s11658-021-00275-8

**Published:** 2021-07-08

**Authors:** An Yang, Xin Liu, Ping Liu, Yunzhang Feng, Hongbo Liu, Shen Gao, Limin Huo, Xinyan Han, Jurong Wang, Wei Kong

**Affiliations:** 1 Handan Central Hospital, Handan, 056001 Hebei Province China; 2 Affiliated Hospital of Hebei University of Engineering, Handan, 056002 Hebei Province China; 3 Handan First Hospital, Handan, 056002 Hebei Province China

**Keywords:** Gastric cancer, lncRNA, UCA1, miR-145, MYO6

## Abstract

**Background:**

Long noncoding RNA (lncRNA), urothelial carcinoma-associated 1 (UCA1) is aberrantly expressed in multiple cancers and has been verified as an oncogene. However, the underlying mechanism of UCA1 in the development of gastric cancer is not fully understood. In the present study, we aimed to identify how UCA1 promotes gastric cancer development.

**Methods:**

The Cancer Genome Atlas (TCGA) and Genotype-Tissue Expression (GTEx) data were used to analyze UCA1 and myosin VI (MYO6) expression in gastric cancer. Western blot and quantitative real-time PCR (QPCR) were performed to test the expression level of the UCA1/miR-145/MYO6 axis in gastric cancer cell lines and tissues. The roles of the UCA1/miR-145/MYO6 axis in gastric cancer in vitro and in vivo were investigated by CCK-8 assay, flow cytometry, siRNAs, immunohistochemistry, and a mouse xenograft model. The targeted relationship among UCA1, miR-145, and MYO6 was predicted using LncBase Predicted v.2 and TargetScan online software, and then verified by luciferase activity assay and RNA immunoprecipitation.

**Results:**

UCA1 expression was higher but miR-145 expression was lower in gastric cancer cell lines or tissues, compared to the adjacent normal cell line or normal tissues. Function analysis verified that UCA1 promoted cell proliferation and inhibited cell apoptosis in the gastric cancer cells in vitro and in vivo. Mechanistically, UCA1 could bind directly to miR-145, and MYO6 was found to be a downstream target gene of miR-145. miR-145 mimics or MYO6 siRNAs could partly reverse the effect of UCA1 on gastric cancer cells.

**Conclusions:**

UCA1 accelerated cell proliferation and inhibited cell apoptosis through sponging miR-145 to upregulate MYO6 expression in gastric cancer, indicating that the UCA1/miR-145/MYO6 axis may serve as a potential therapeutic target for gastric cancer.

## Background

Gastric cancer (GC) is the fifth most prevalent cancer and the third leading cause of cancer-related mortality worldwide [1]. Despite emerging therapies including immunotherapy and targeted therapy for treatment of GC, the prognosis of patients with GC is still far from satisfactory, owing to most patients being diagnosed at an advanced stage, when treatment is not effective [2]. Therefore, further insights into the molecular mechanisms underlying GC development may help discover potential therapeutic targets.

Long noncoding RNAs (lncRNAs) are RNA transcripts longer than 200 nucleotides and lacking protein-coding potential, which play critical roles in numerous biological processes, including cancer development. Accumulating evidence has demonstrated that lncRNAs function as tumor suppressor genes or oncogenes to influence cancer cell proliferation, apoptosis, invasion, or migration [3]. The lncRNA, urothelial carcinoma associated 1 (UCA1), located at the human chromosome 19p13.12, was discovered in bladder cancer. Recently, some studies have shown that UCA1 is abnormally expressed in many cancers, for example, colorectal carcinoma, cervical carcinoma, and GC [4–6]. Although previous reports have indicated that high-UCA1 expression might exert an oncogenic effect in the pathogenic process of GC and act as a diagnostic and prognostic biomarker [7], the precise pathophysiological functions and detailed signaling pathways of UCA1 in GC remain to be determined.

LncRNAs are enriched and stable in exosomes, which are small membrane-derived vesicles [8]. Exosomes could regulate tumor proliferation and metastasis as cell-to-cell mediators by transferring transcripts, including lncRNAs, microRNAs, and mRNAs [9]. Emerging evidence has demonstrated that lncRNAs could cooperate with miRNAs to facilitate tumorigenesis by post-transcriptional regulation [10, 11]. It has been reported that UCA1 could function as a miR-495 or miR-203 sponge to regulate PRL-3 or ZEB2 expression in GC [12, 13]. However, the UCA1-miRNA-mRNA communication in GC remains largely unidentified.

In this study, we observed that serum exosomal UCA1 was overexpressed in GC patients compared to the healthy subjects, and UCA1 expression was higher in GC tissues than that in the adjacent normal tissues. Furthermore, UCA1 could interact directly with miR-145 and act as a sponge of miR-145 to regulate MYO6 expression. We further found that the UCA1-miR145-MYO6 pathway plays a vital role in GC cell proliferation and apoptosis, which may be valuable as a potential remedial target in GC.

## Materials and methods

### Patients and collection of samples

The GC tissues and paired adjacent normal tissues were collected from 86 GC patients after surgical resection from 2017 to 2019 in the Department of General Surgery of Handan Central Hospital. Patients did not undergo radiotherapy, chemotherapy, or any other treatments before surgery. Half of each tissue was immediately snap-frozen in liquid nitrogen and stored at – 80 °C for further experiments. The remaining part of each tissue was processed by formalin fixation and paraffin embedment. Blood samples were obtained from 85 GC patients and 50 healthy controls. We performed the study following the principles of the Declaration of Helsinki. The study was approved by Handan Central Hospital Ethics Committee (approval no. 20160415007, date: 2016.5.22). Written informed consent was obtained from all patients.

### Isolation of exosomes from serum

Following the manufacturer’s instructions, serum exosomes were isolated using the Exosome Isolation Reagent for plasma or serum (RiboBio, C10110-2, Guangzhou, China). Exosome pellets were resuspended in phosphate-buffered saline (PBS). The concentration of exosomes was determined by the BCA Protein Assay Kit (Tiangen Biotech, Beijing, China).

### Transmission electron microscopy

The exosomes were diluted in PBS to 0.5 mg/ml. Subsequently, the exosomes were loaded onto the carbon-coated copper mesh on the filter paper. After 5 min of drying, 1% phosphotungstic acid was dropped to stain exosomes, and then exosomes were dried for 10 min. Finally, the morphology of exosomes was viewed under a Hitachi H-7500 transmission electron microscope (TEM) (Hitachi, Japan).

### Dynamic light scattering

The exosomes were diluted in DPBS to 0.1 μg/μl. After the equilibrium of the dynamic light scattering (DLS) device had been stabilized (DynaPro NanoStar, USA), the exosome samples were transferred into the sample chamber to start measurement at 20 °C with the 633 nm laser at an angle of 173°. Each sample was read three times.

### The Cancer Genome Atlas database analysis

The TIMER2 and GEPIA2 websites were used to observe the expression difference of UCA1 and MYO6 between tumor and adjacent normal tissues of The Cancer Genome Atlas (TCGA) data and the Genotype-Tissue Expression (GTEx) data.

### Cell lines, culture conditions and cell transfection

The human GC cell lines BGC-823, MGC-803, AGS, SGC-7901 and the human normal gastric epithelial cell line GES-1 were obtained from the National Infrastructure of Cell Line Resource (Beijing, China). Cells were cultured in RPMI 1640 or DMEM supplemented with 10% fetal bovine serum, 100 U/mL penicillin, and 100 mg/mL streptomycin (Invitrogen, USA) in the incubator at 37℃ with 5% CO_2_. Cell transfection was performed with Lipofectamine 3000, in accordance with the manufacturer’s instructions (Invitrogen, USA).

### Construction of plasmids

The full-length UCA1 cDNA sequence obtained by PCR was inserted into the pcDNA-3.1 vector to generate the UCA1 overexpression plasmid, named pcDNA3.1-UCA1. si-UCA1, si-MYO6, siRNA negative control (si-NC), miR-145 mimic, and miR-145 mimic negative control (miR-NC) were obtained from GenePharma (Shanghai, China). The sequence of binding sites between UCA1 and miR-145 was determined by the RNAhybrid online software (http://bibiserv2.cebitec.uni-bielefeld.de/rnahybrid), and the binding sites between MYO6 and miR-145 were predicted by TargetScan. The UCA1 WT/MUT and MYO6 3ʹ-UTR WT/MUT sequences were inserted into the pGL3 vector.

### Cell proliferation

Cell Counting Kit-8 (CCK-8, Beyotime, Shanghai, China) was used to observe cell proliferation. Briefly, after transfection for 48 h, different kinds of cells were seeded into 96-well plates. On day 1, 2, 3, cells were incubated in CCK-8 solution for one hour. Then the optical density (OD) was tested by a microplate reader at 450 nm. The experiments were repeated three times.

### Flow cytometry

Transfected cells were harvested and stained with Annexin V-FITC/PI (BD Biosciences) in accordance with the manufacturer’s instructions. Then cells were analyzed by a FACScan flow cytometer and Cell Quest software (BD, Biosciences). For cell-cycle analysis, cells were stained with propidium iodide (PI, Sigma), then analyzed by a FACScan flow cytometer and Cell Quest software. The number of cells in the G0/G1, S, or G2/M phase was determined and expressed as a percentage.

### RNA extraction and quantitative real-time PCR assay

TRIzol reagent (Invitrogen, USA) was used for total RNA isolation from exosomes, tissues or cells. First-strand cDNA was synthesized from total RNA using a Takara PrimeScript RT Reagent Kit. Quantitative real-time PCR (Q-PCR) was performed using a Takara SYBR Green PCR Kit. The relative gene expression was calculated using the 2^−ΔΔCT^ method. Results were normalized to the expression of GAPDH or U6. The PCR primer sequences were as follows: UCA1 forward: 5ʹ- CTCTCCTATCTCCCTTCACTGA-3ʹ, reverse: 5ʹ-CTTTGGGTTGAGGTTCCTGT-3ʹ; MYO6 forward: 5ʹ- CAGAGCAACGTGCTCCAAAGTC-3ʹ, reverse: 5ʹ- GAAGCGTTGCTG TCGGTTCA; GAPDH forward: 5ʹ- CACCATTGGCAATGAGCGGTTC-3ʹ, reverse: 5ʹ- AGGTCTTTGCGGATGTCCACGT-3ʹ; miR-145-5p forward: 5ʹ-ACACTCCAGCTGGGTCCCTAAGGACCCTTTT-3ʹ, reverse: 5ʹ- CTCAACTGGTGTCGTGGAGTCGGCAATTCAGTTGAGCAGGTCAA-3ʹ; U6‐forward: 5ʹ-CTCGCTTCGGCAGCACATA-3ʹ, reverse: 5ʹ-AACGATTCACGAATTTGCGT-3ʹ.

### Western blotting

Total proteins were extracted from exosomes, tissues, or cells using radioimmunoprecipitation assay buffer. The protein concentration was measured by BCA assay. 40 μg of proteins of each sample were separated by SDS-PAGE and then transferred to PVDF membrane. The membrane was blocked for 1 h with 5% non-fat milk in TBST at room temperature and incubated with the corresponding primary antibodies: anti-TSG101 (Abcam, UK), anti-CD63 (Abcam, UK), anti-GM130 (Abcam, UK), anti-MYO6 (Abcam, UK), and anti-GAPDH (Abcam, UK) at 4 °C overnight, and then incubated with appropriate secondary antibodies at room temperature for 1 h. Finally, specific protein bands were visualized by an enhanced chemiluminescence detection kit.

### Immunohistochemistry

For each sample, paraffin-embedded sections (5 μm) were prepared for immunohistochemistry (IHC). After dewaxing, rehydration, heated antigen retrieval, and blocking, slides were incubated at 4℃ overnight with primary antibodies and then incubated at room temperature for 30 min with biotin-labeled secondary antibodies. PBS was used for the negative control instead of primary antibodies. Diaminobenzidine was used for chromogenic revelation. The images were observed under a microscope.

### RNA immunoprecipitation assay

According to the manufacturer’s instructions, the RNA immunoprecipitation (RIP) experiment was performed using an EZ-Magna Rip Kit (Millipore, USA). In brief, cells were harvested and lysed with RIP lysis buffer containing RNase inhibitors and proteinase inhibitors. Then the lysates were incubated with RIP buffer containing magnetic beads conjugated with human anti-Ago2 antibody or normal mouse IgG control. The RNAs in the immunoprecipitation were purified and analyzed by Q-PCR.

### Luciferase reporter assay

GC cells were co-transfected with UCA1 or MYO6 WT/MUT luciferase reporter plasmids and miR-145 mimics or miR-NC using Lipofectamine 2000. Luciferase activity was detected with the dual-luciferase assay system (Promega). Relative luciferase activity was normalized to the Renilla activity. Each experiment was performed twice in triplicate.

### Tumor xenograft experiments

The shRNA targeting UCA1 (shUCA1) or negative control (shNC) was inserted into the pLKO.1 vector to construct the shUCA1 or shNC plasmid, followed by transfection into MGC-803 cells, which were screened by puromycin selection for 7 days.

BALB/c male nude mice aged 4–6 weeks were bought from Beijing Vital River Laboratory Animal Technology Company and were housed under standard conditions. 5 × 10^6^ cells stably expressing either shUCA1 or shNC were subcutaneously injected into the right rear flank of nude mice. Tumor size was observed over time and measured every 7 days. Four weeks after injection, mice were euthanized and the xenografts were collected for subsequent experiments. The animal experiment protocol complied with the international guidelines and was approved by the Ethics Committees of Handan Central Hospital (approval no. 20160415007, date: 2016.5.22).

### Statistical analysis

Results are presented as mean ± standard deviation (SD). The difference between the two groups was compared by the two-tailed Student’s t-test. One-way ANOVA was applied to measure the differences among more than two groups. Statistical analyses were performed using SPSS 20.0 software. P < 0.05 was considered to be statistically significant.

## Results

### UCA1 is enriched in serum exosomes from patients with GC

**Fig. 1:**
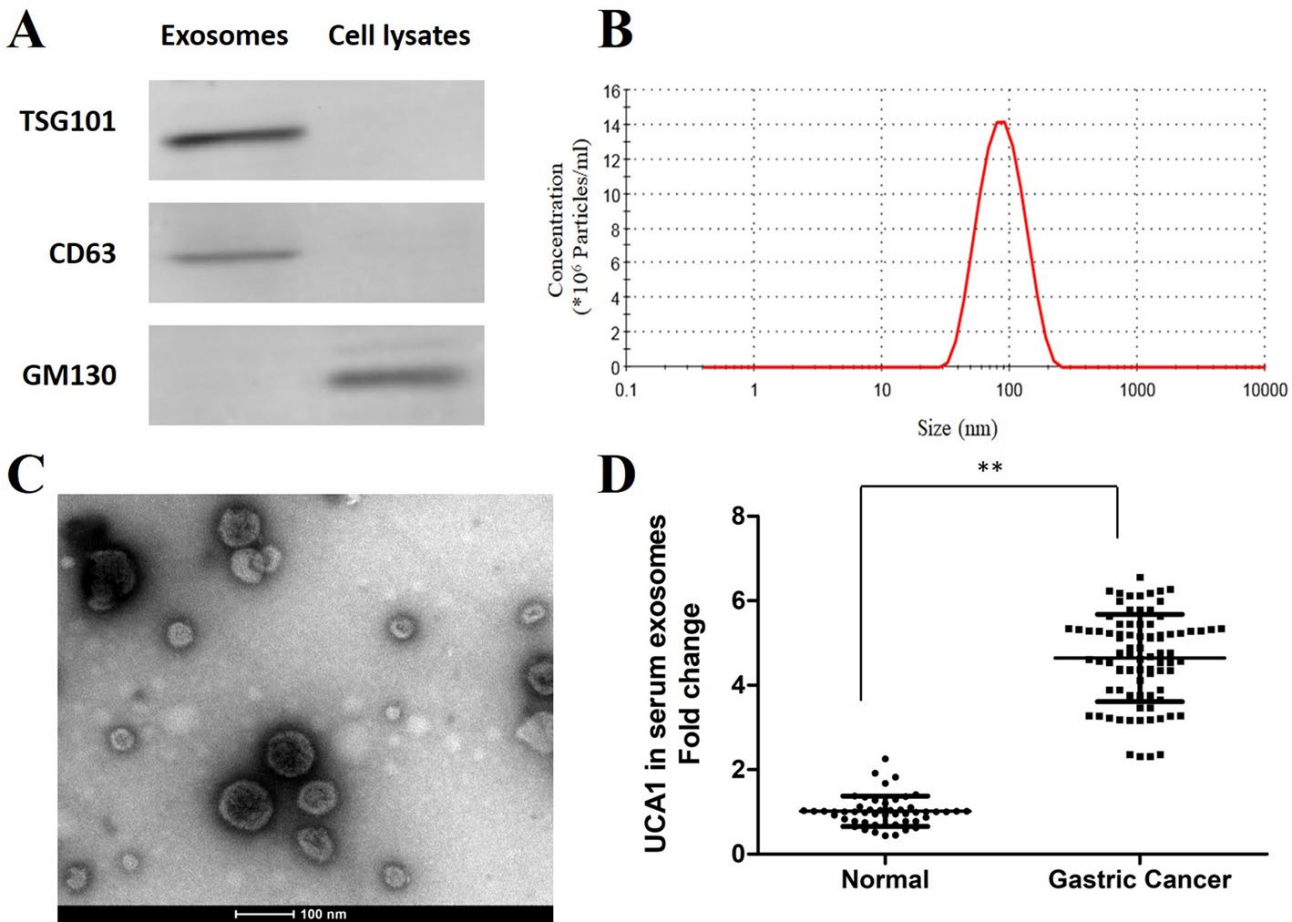
UCA1 expression increased in serum exosomes from GC patients. **A** Western blot results showing the exosomal markers (TSG101, CD63, and GM130) expressed in serum exosomes of GC patients. **B** DLS results showing the exosomes size distribution. **C** Representative image of exosomes detected by TEM. **D** Q-PCR results for the UCA1 expression level in serum exosomes from GC patients and normal individuals. **p < 0.01 in comparison with normal individuals

In this study, serum samples from 85 GC patients and 50 healthy individuals were collected, and then exosomes were isolated from the serum. We identified these vesicles using Western blot. It confirmed that two well-known exosomal markers, TSG101 and CD63, were positively expressed. It also showed that GM130 was negatively expressed in exosomes (Fig. 1A). As a negative control, the negative expression of GM130 identified no cellular contamination in the extract exosomes. TEM and DLS revealed that plasma extract exosomes have a spherical-shaped morphology and 80–135 nm was the peak size range (Fig. 1B, C). To identify UCA1 expression in exosomes, we extracted serum exosomal RNAs from GC patients and healthy individuals. Then, the Q-PCR assay confirmed that UCA1 was enriched in serum exosomes of GC patients, compared with normal people (Fig. 1D).

### UCA1 is overexpressed in GC tissues and cell lines

**Fig. 2:**
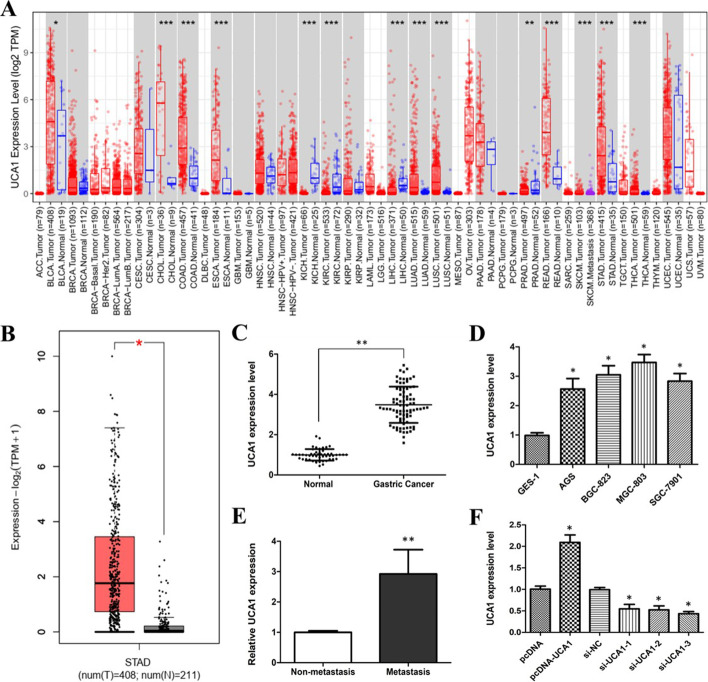
UCA1 expression was upregulated in GC tissues and cell lines. **A** UCA1 expression in different cancers was analyzed by TIMER2. **B** The box plot data about UCA1 expression were supplied from TCGA and GTEx databases. **C** UCA1 expression level in GC tissues and adjacent normal tissues determined by Q-PCR. **D** Q-PCR results displaying UCA1 expression level in GC cell lines and human normal gastric epithelium cell line GES-1. **E** Q-PCR showing UCA1 expression level in non-metastatic and metastatic GC tissues. **F** UCA1 overexpression or knockdown by transfecting pcDNA3.1-UCA1 or siUCA1. *p < 0.05, **p < 0.01, ***p < 0.001, compared with normal tissues or cell lines

We applied the TIMER2 approach to analyze the expression status of UCA1 across various types of cancer of the TCGA data. As shown in Fig. 2A, UCA1 expression in many kinds of tumor tissues is higher than that in the corresponding control tissues, such as bladder urothelial carcinoma (BLCA), cholangiocarcinoma (CHOL), lung squamous cell carcinoma (LUSC), stomach adenocarcinoma (STAD), and thyroid carcinoma (THCA). Next, we further analyzed the UCA1 expression in the TCGA data and the GTEx data using the GEPIA2 approach. It showed that the expression level of UCA1 in 408 STAD tissues was obviously higher than that in 211 normal tissues (Fig. 2B).

To further observe UCA1 expression level in GC tissues, the Q-PCR assay was performed and the results are shown in Fig. 2C. UCA1expression level in GC tissues was higher than that in normal tissues. Similarly, compared with the human normal gastric epithelial cell line GES-1, human GC cell lines (AGS, BGC-823, MGC-803, and SGC-7901) expressed higher levels of UCA1 (Fig. 2D). Although the differences in UCA1 expression level among these cell lines did not reach statistical significance, MGC-803 cells expressed the highest level of UCA1. This might be because these cell lines derived from different origins and had different differentiation properties.

We also investigated the relationship between UCA1 expression and clinical features of GC. The 85 GC patients were assigned to the non-metastasis and metastasis group, or low (n = 46) and high (n = 39) expression groups based on the median expression level. Figure 2E and Table 1 show that the UCA1 expression level was correlated with tumor size, metastasis, and recurrence. These results indicated that UCA1 upregulation may be involved in GC progression and metastasis.

**Table 1:** Relationship between UCA1 expression and clinical features of gastric cancer

Clinical features		Patients (n = 85)	UCA1 expression		*X*^*2*^	*P*
			Low (n = 46)	High (n = 39)		
Age	≤ 60	47	25 (53.19%)	22 (46.81%)	0.036	0.849
	> 60	38	21 (55.26%)	17 (44.74%)		
Gender	Male	50	26 (52.00%)	24 (48.00%)	0.219	0.640
	Female	35	20 (57.14%)	15 (42.86%)		
Tumor size	≤ 5 cm	43	28 (65.12%)	15 (34.88%)	4.240	0.039
	> 5 cm	42	18 (42.86%)	24 (57.14%)		
Metastasis	Absent	43	29 (67.44%)	14 (32.56%)	6.222	0.013
	Present	42	17 (40.48%)	25 (59.52%)		
Recurrence	Absent	40	27 (67.50%)	13 (32.50%)	5.449	0.020
	Present	45	19 (42.22%)	26 (57.78%)		

### UCA1 promotes GC cell proliferation in vitro

To explore whether UCA1 could regulate GC cell proliferation, loss- or gain-of-function experiments were performed. The pcDNA3.1-UCA1 plasmid was constructed and transfected into the MGC 803 cell line to induce ectopic overexpression of UCA1. Three UCA1 specific siRNAs were designed and transfected into the MGC 803 cell line to knock down the expression of UCA1. UCA1 overexpression or knockdown was verified by Q-PCR (Fig. 2F). The expression level of UCA1 was increased in the cells transfected with pcDNA3.1-UCA1 but decreased by si-UCA1. It also showed that si-UCA1-3 had the highest interference efficiency, so si-UCA1-3 was selected for the subsequent experiments.

**Fig. 3:**
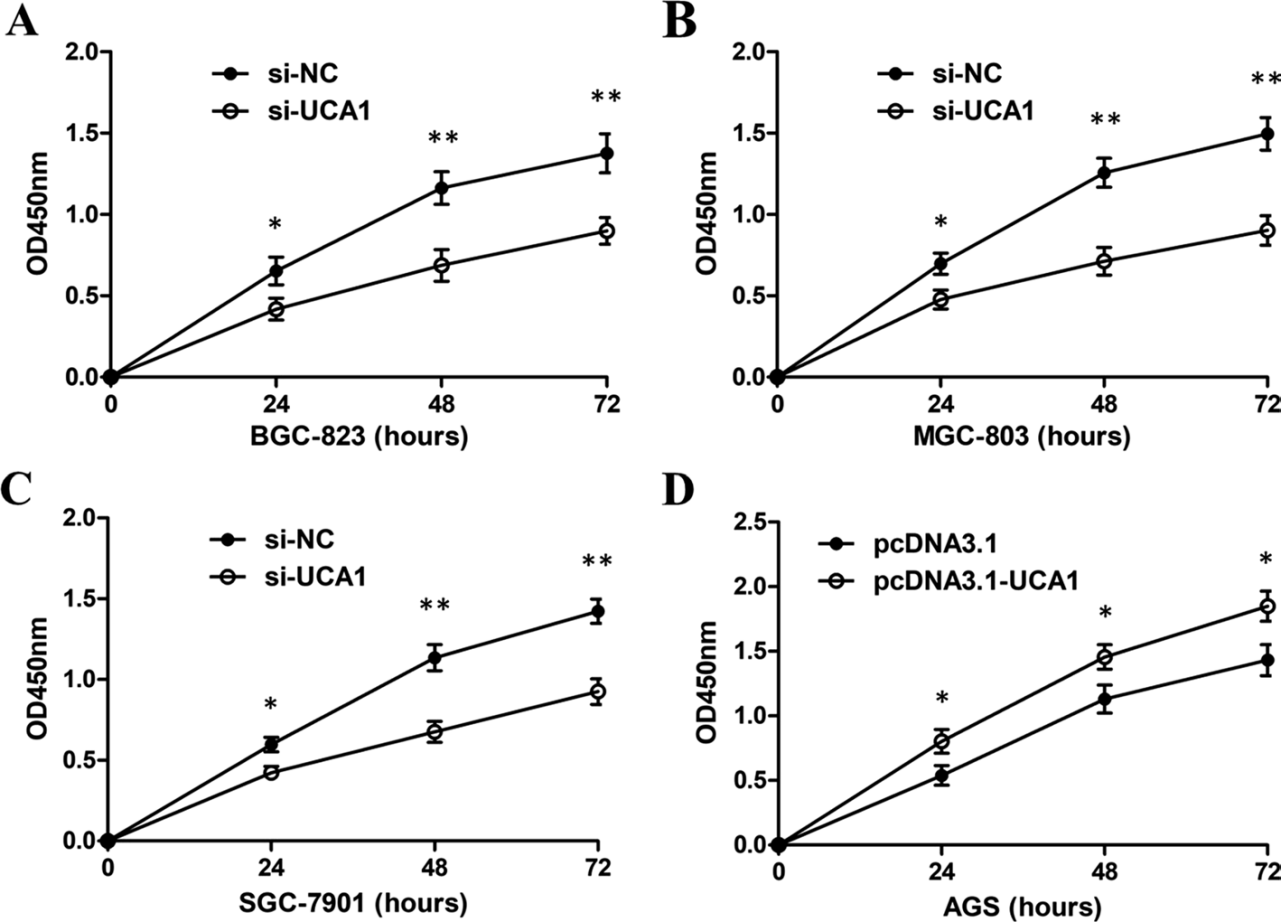
UCA1 promotes GC cell proliferation in vitro. **A** CCK-8 experiment indicated that UCA1 knockdown inhibited cell proliferation of BGC-823 cells. **B** CCK-8 assay revealed that UCA1 knockdown inhibited MGC-803 cell proliferation. **C** CCK-8 assay showed that UCA1 knockdown inhibited SGC-7901 cell proliferation. **D** CK-8 assay showed that UCA1 overexpression promoted AGS cell proliferation. *p < 0.05, **p < 0.01, compared with control cells

The CCK-8 assay confirmed that UCA1 knockdown induced by si-UCA1 remarkably suppressed BGC-823, MGC-803, and SGC-7901 cell proliferation (Fig. 3A–C). Moreover, UCA1 overexpression induced by pcDNA3.1-UCA1 significantly promoted AGS cell proliferation (Fig. 3D).

### UCA1 knockdown promotes G1 arrest and causes apoptosis in GC cells

**Fig. 4:**
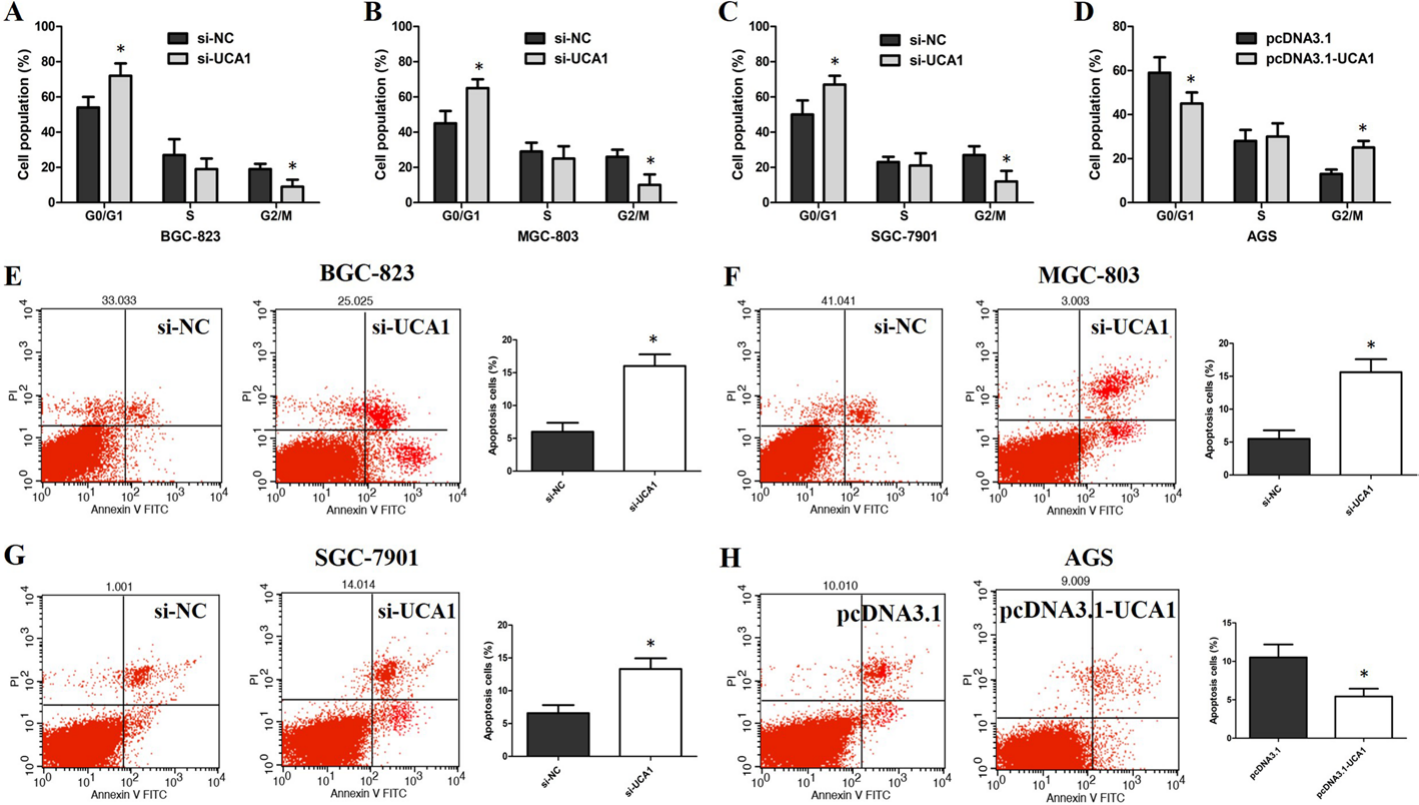
UCA1 downregulation causes G1 arrest and apoptosis in GC cells in vitro. **A** Flow cytometry assay showed that BGC-823 cells transfected with siUCA1 had G0/G1 phase arrest. **B** Flow cytometry assay showed that MGC-803 cells transfected with siUCA1 had G0/G1 phase arrest. **C** Flow cytometry assay showed that SGC-7901 cells transfected with siUCA1 had G0/G1 phase arrest. **D** Flow cytometry assay showed that AGS cells transfected with pcDNA3.1-UCA1 decreased the G0/G1 phase cell percentage and increased the G2/M phase cell percentage. **E** Flow cytometry assay showed that BGC-823 cells transfected with siUCA1 had a higher apoptotic rate. **F** Flow cytometry assay showed that MGC-803 cells transfected with siUCA1 had a higher apoptotic rate. **G** Flow cytometry assay showed that SGC-7901 cells transfected with siUCA1 had a higher apoptotic rate. **H** Flow cytometry assay showed that AGS cells transfected with pcDNA3.1-UCA1 had a lower apoptotic rate. *p < 0.05, compared with control cells

We further observed the roles of UCA1 in the GC cell cycle using flow cytometry. The results showed that UCA1 knockdown caused G1-phase arrest of BGC-823, MGC-803, and SGC-7901 cells (Fig. 4A–C), but UCA1 upregulation decreased the G0/G1 phase percentage and increased the G2/M phase percentage of AGS cells (Fig. 4D).

Moreover, we also observed the roles of UCA1 in GC cell apoptosis using flow cytometry. The results indicated that the apoptotic percentage of BGC-823, MGC-803, and SGC-7901 cells was notably increased when UCA1 was downregulated (Fig. 4E–G), whereas the cell apoptosis was obviously decreased in AGS cells induced by UCA1 overexpression (Fig. 4H). These results indicated that UCA1 inhibited GC cell apoptosis. The effect of UCA1 on promoting GC cell proliferation might be through regulating the cell cycle and apoptosis.

### UCA1 knockdown restrains GC tumor growth in vivo

**Fig. 5:**
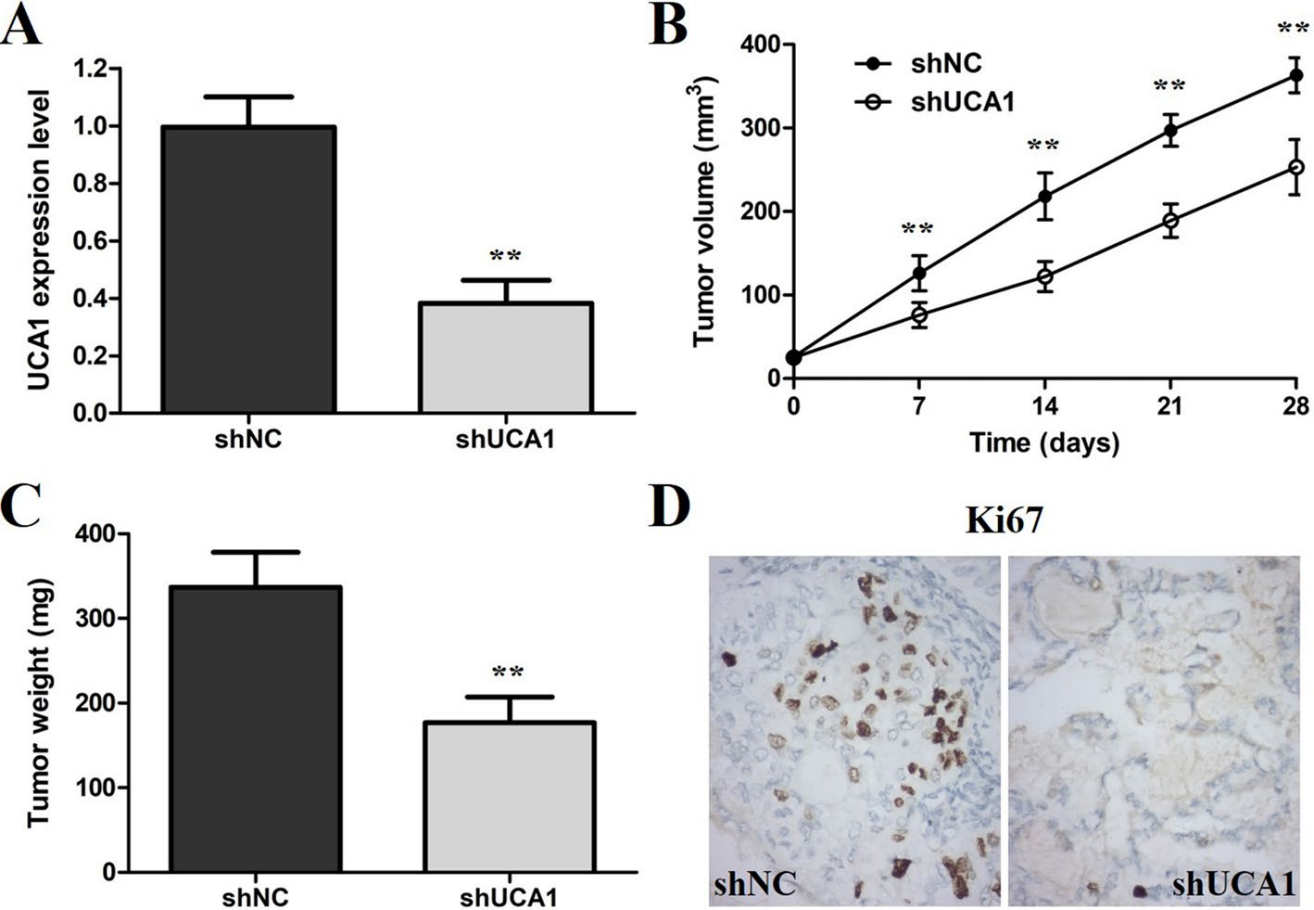
UCA1 knockdown inhibited tumor growth of GC in vivo. **A** Q-PCR verified the knockdown effect of shUCA1. **B** The tumor volume curve of nude mice was drawn and UCA1 knockdown inhibited MGC-803 tumor growth in vivo. **C** Tumor weights of nude mice were recorded. **D** IHC revealed that xenograft tumors derived from MGC-803 cells with UCA1 knockdown had lower expression of Ki67. **p < 0.01, compared with the control group

To confirm whether UCA1 was involved in GC tumorigenesis in vivo, MGC-803 cells stably transfected with shUCA1 or shNC were inoculated into nude mice. The UCA1 expression level was confirmed by Q-PCR assay using xenograft tumor tissues. It revealed that the expression level of UCA1 in the tumor tissues from the shUCA1 group was significantly lower than that in the shNC group (Fig. 5A). The tumor growth rate was notably slower in the shUCA1 group than that in the shNC group (Fig. 5B). Moreover, the average tumor weight was significantly higher in the shNC group than that in the shUCA1 group (Fig. 5C). The results of IHC showed that the tumor tissues derived from the shUCA1 group expressed weaker staining of Ki67 than that in the shNC group (Fig. 5D). These outcomes suggest that UCA1 might play a crucial role in promoting the tumor growth of the GC in vivo.

### UCA1 inhibits miR-145 expression in GC cells

**Fig. 6:**
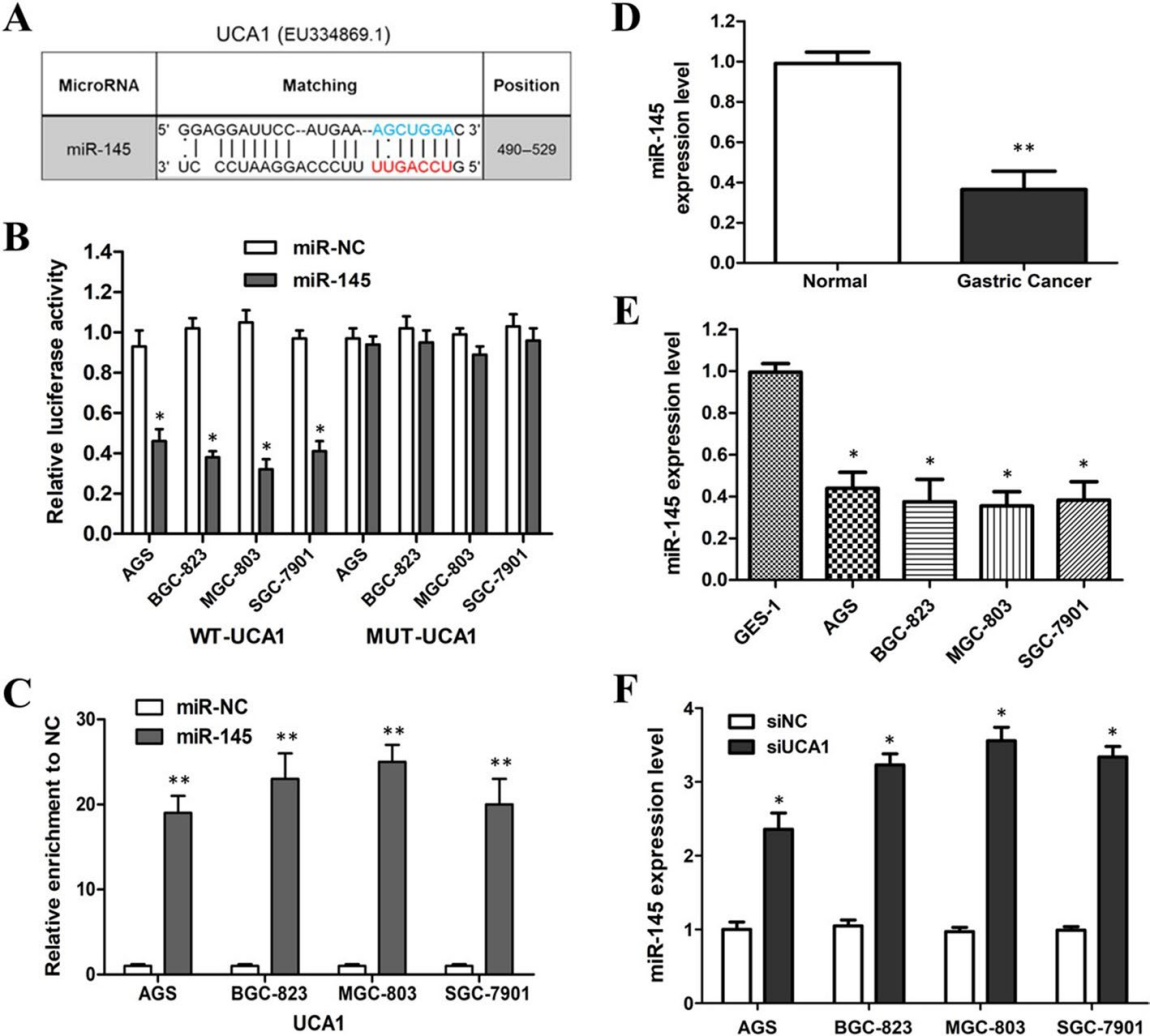
UCA1 acts as a sponge of miR-145 in GC. **A** Putative binding site of miR-145 on UCA1. The red nucleotides were the seed sequences of miR-145 and the blue nucleotides were deleted in the MUT-UCA1. **B** Dual-luciferase reporter assays of GC cells co-transfected with WT/MUT UCA1 and miR-145 mimics or miR-NC. **C** RIP assay showed that both UCA1 and miR-145 existed in the production precipitated by anti-AGO2. **D** miR-145 expression levels in GC tissues and normal tissues. **E** miR-145 expression levels in GC cell lines. F. Knockdown of UCA1 increased the expression level of miR-145 IN GC cell lines. *p < 0.05, **p < 0.01, compared with control cells

Because interacting with miRNAs is an important way that lncRNAs can exert their function, we further examined the interaction between UCA1 and miRNAs in GC cells. The RNAhybrid software program was applied to predict the potential miRNAs that could interact with UCA1. It predicted that miR-145 could bind to UCA1. Figure 6A shows the putative binding sites. In order to verify the interaction between miR-145 and UCA1, we constructed WT-UCA1 or MUT-UCA1 luciferase reporter plasmids and performed luciferase reporter gene assays. The results indicated that WT-UCA1 and miR-145 mimic co-transfection greatly decreased the luciferase activities, while the luciferase activities in the MUT-UCA1 and miR-145 mimics co-transfection group showed no obvious change (Fig. 6B). RIP assay confirmed that miR-145 and UCA1 simultaneously existed in the precipitation, suggesting that miR-145 could directly bind to UCA1 (Fig. 6C).

Figure 6D, E showed that the expression level of miR-145 was decreased in GC cells and tissues, which is opposite to the UCA1 expression in GC cells and tissues. In addition, miR-145 expression was increased in GC cells when UCA1 was knocked down by siRNAs (Fig. 6F). These outcomes indicated that UCA1 could sponge miR-145 to inhibit its biological function.

### MYO6 is a direct target of miR-145 in GC cells

**Fig. 7:**
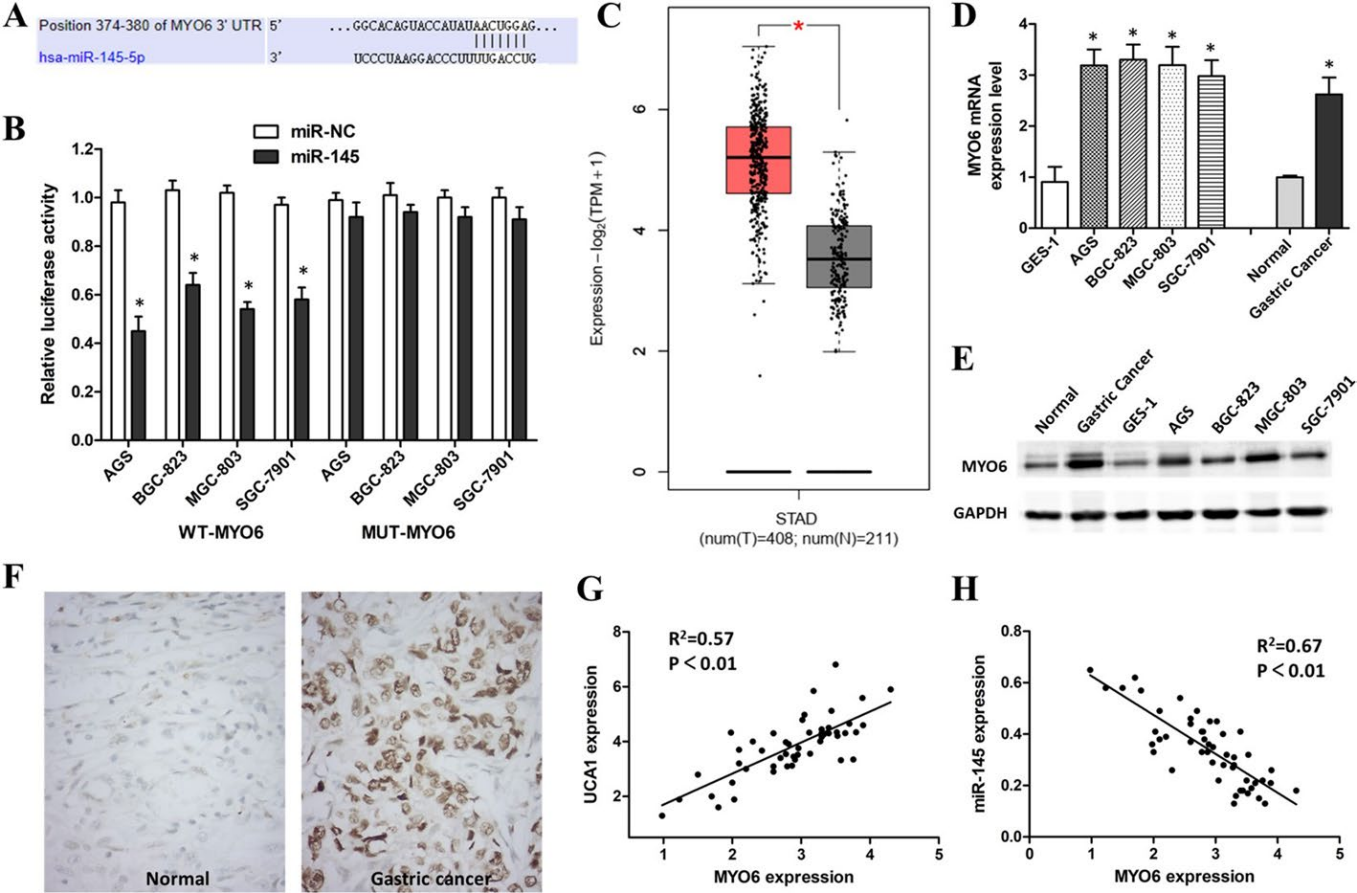
MYO6 is a downstream target of miR-145. **A** Binding sites between MYO6 and miR-145 were predicted by TargetScan 7.2. **B** Dual-luciferase reporter assays of GC cells co-transfected with WT/MUT MYO6 and miR-145 mimics or miR-NC. **C** Box plot data about UCA1 expression were obtained from TCGA and GTEx databases. **D** Q-PCR showing MYO6 expression level in GC cell lines and tissues. **E** Western blot showing MYO6 expression level in GC tissues and cell lines. **F** IHC showing MYO6 expression level in GC tissues. **G** Scatter diagram showing a positive correlation of UCA1 and MYO6 in GC tissues by Q-PCR. **H** Scatter diagram showing a negative correlation of miR-145 and MYO6 in GC tissues by Q-PCR. *p < 0.05, compared with control cells or tissues

TargetScan 7.2 was utilized to explore potential target genes of miR-145, and we found that MYO6 was a candidate target of miR-145 (Fig. 7A). Luciferase reporter gene assays confirmed that miR-145 mimics had no effect on the MUT-MYO6 group in GC cells, but greatly reduced the luciferase activity in the WT-MYO6 group (Fig. 7B). These results suggested that MYO6 is a target of miR-145.

The COAD data from the TCGA database showed that MYO6 was overexpressed in 408 STAD tissues (Fig. 7C). The mRNA and protein expression levels of MYO6 in GC cells and tissues were higher than those in normal cells and tissues (Fig. 7D, E). IHC results also showed that GC tissues expressed higher levels of MYO6 than normal tissues (Fig. 7F). Furthermore, we found that MYO6 expression levels were negatively correlated with miR-145 but positively correlated with UCA1 in GC tissues by Q-PCR (Fig. 7G, H).

### miR-145 regulates GC cell proliferation, cell cycle, and apoptosis by targeting MYO6

**Fig. 8:**
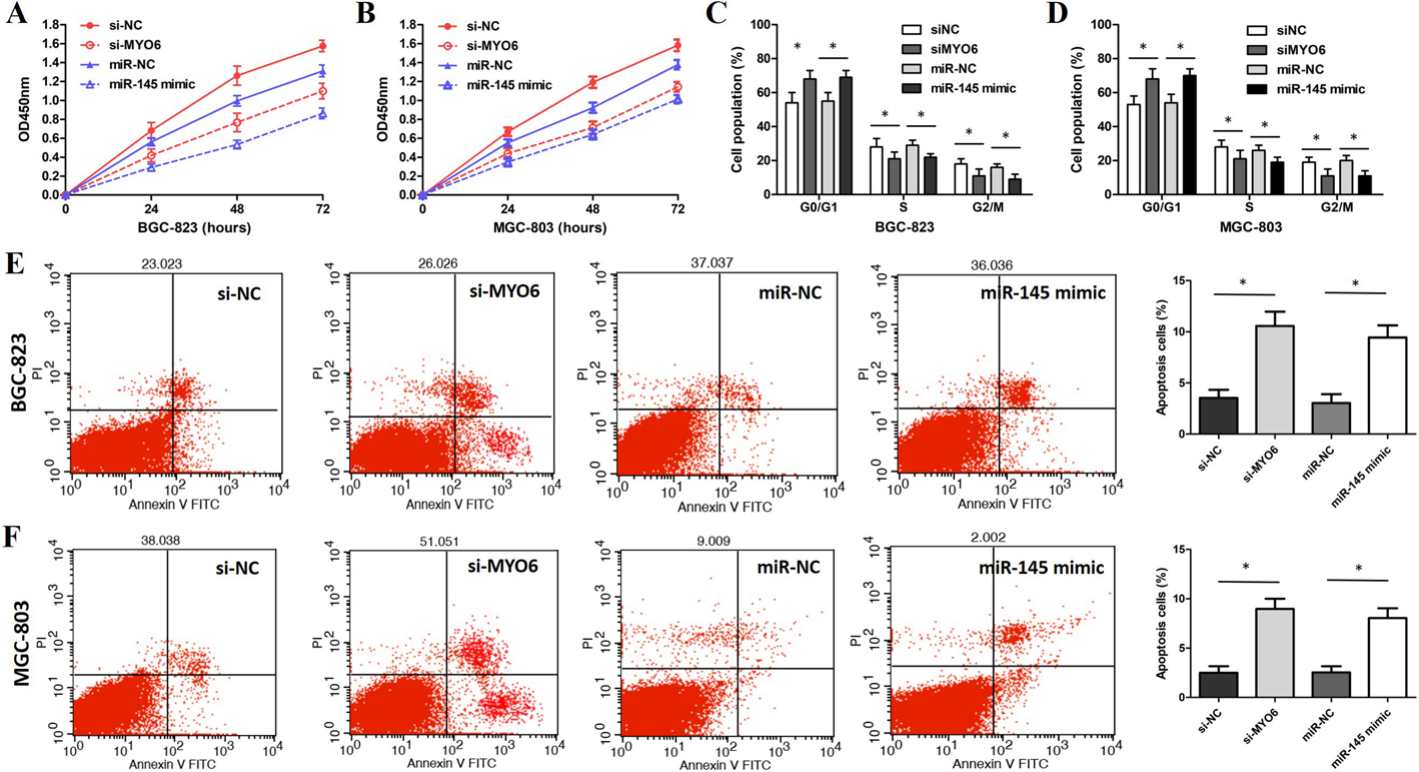
miR-145/MYO6 axis regulates cell proliferation and apoptosis of GC cells. **A** CCK-8 assay showed that MYO6 knockdown or miR-145 mimics inhibited cell proliferation of BGC-823 cells. **B** CCK-8 assay showed that MYO6 knockdown or miR-145 mimics inhibited cell proliferation of MGC-803 cells. **C** BGC-823 cells transfected with siMYO6 or miR-145 mimics induced G0/G1/ phase arrest. **D** Flow cytometry assay showed that MGC-803 cells transfected with siMYO6 or miR-145 mimics had G0/G1 phase arrest. **E** Flow cytometry assay showed that BGC-823 cells transfected with siUCA1 or miR-145 mimics had a higher apoptotic rate. **F** Flow cytometry assay verified that UCA1 knockdown in MGC-803 cells by siUCA1 transfection induces apoptosis. *p < 0.05, compared with control cells

To confirm the effect of miR-145 and MYO6 on the GC cells’ growth, cell cycle, and apoptosis, siMYO6 and miR-145 mimics were used for the knockdown of MYO6 in GC cells. The CCK-8 results revealed that MYO6 downregulation induced by siMYO6 or by miR-145 mimics inhibited the proliferation of BGC-823 and MGC-803 cells (Fig. 8A, B). Flow cytometry results showed that MYO6 knockdown by siMYO6 or miR-145 mimics caused G1-phase cell-cycle arrest of BGC-832 and MGC-803 cells (Fig. 8C, D). Moreover, siMYO6 or miR-145 mimics significantly increased the cell apoptosis of BGC-832 and MGC-803 cells (Fig. 8E, F).

### UCA1 partially reverses the effects of the miR-145/MYO6 pathway on GC cell proliferation and apoptosis

**Fig. 9:**
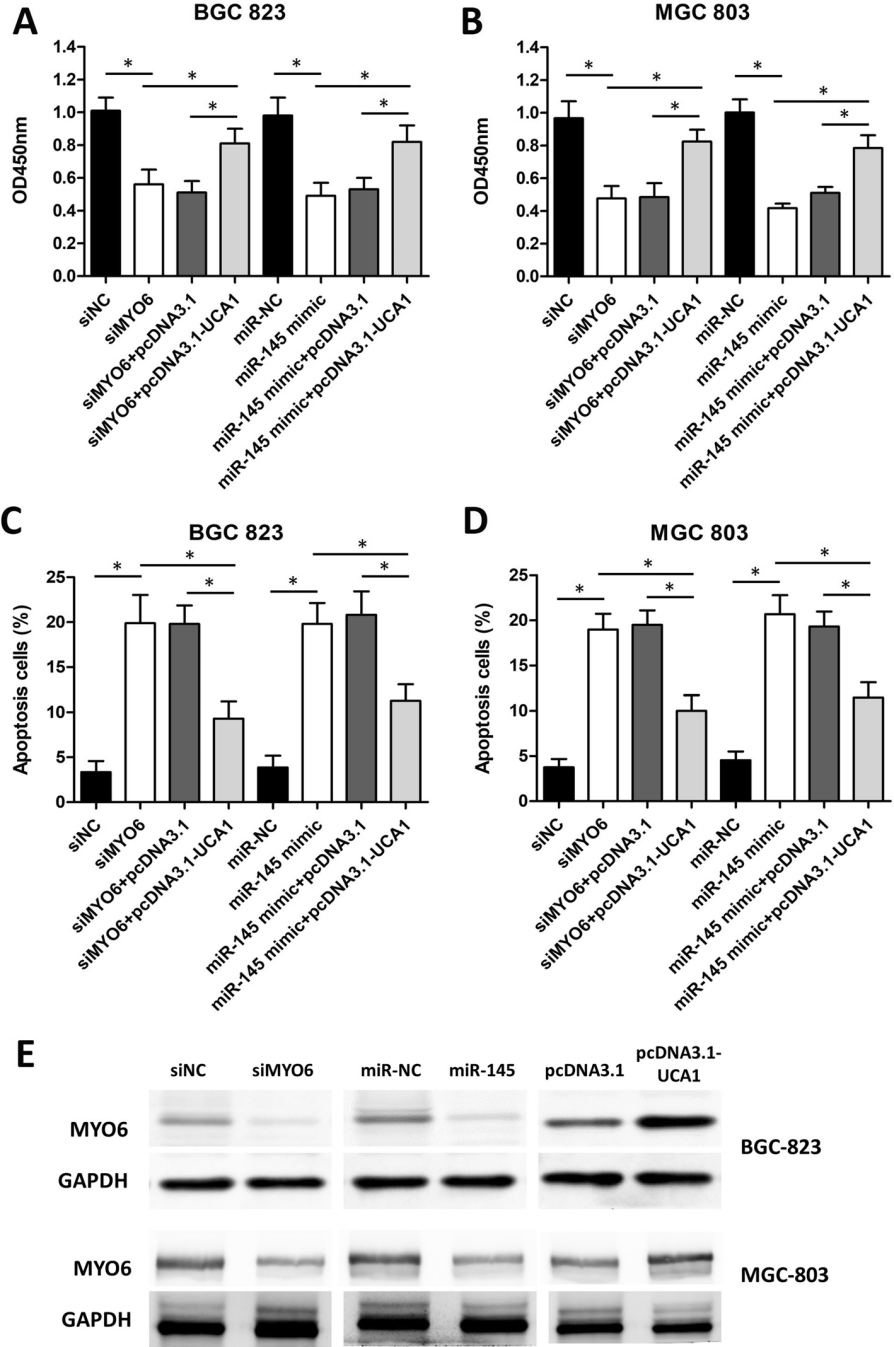
UCA1 reverses the effect of the miR-145/MYO6 axis on GC cell proliferation and apoptosis. **A** CCK-8 assay showed that pcDNA3.1-UCA1 partially reversed the repression effect of miR-145 mimics or siMYO6 on BGC-823 cell proliferation. **B** CCK-8 assay showed that pcDNA3.1-UCA1 partially reversed the repression effect of miR-145 mimics or siMYO6 on MGC-803 cell proliferation. **C** Flow cytometry assay showed that the increased apoptosis induced by miR-145 mimics or siMYO6 in BGC-823 cell lines was suppressed by pcDNA3.1-UCA1 co-transfection. **D** Flow cytometry assay showed that the increased apoptosis induced by miR-145 mimics or siMYO6 in MGC-803 cell lines was suppressed by pcDNA3.1-UCA1 co-transfection. **E** Western blot results showing MYO6 expression in BGC-823 and MGC-803 cells after siMYO6, miR-145 mimics, or pcDNA3.1-UCA1 transfection. *p < 0.05, compared with control cells

To verify whether UCA1 regulates GC cell proliferation and apoptosis through the miR-145/MYO6 axis, pcDNA3.1-UCA1 was co-transfected with miR-145 mimics or siMYO6 into BGC-832 and MGC-803 cells. The CCK-8 results revealed that UCA1 overexpression induced by pcDNA3.1-UCA1 partially reversed the repression effect of miR-145 mimics or siMYO6 on BGC-823 and MGC-803 cell proliferation (Fig. 9A, B). Moreover, the increased apoptosis induced by miR-145 mimics or siMYO6 in BGC-823 and MGC-803 cell lines was suppressed by pcDNA3.1-UCA1 co-transfection (Fig. 9C, D). The above results suggested that the effects of the miR-145/MYO6 axis on GC cell proliferation and apoptosis were at least partially regulated by UCA1.

## Discussion

**Fig. 10:**
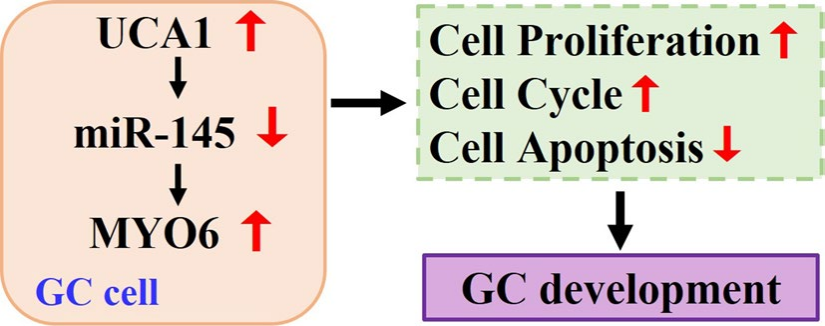
Schematic representation of UCA1 mediated GC progression through miR-145/MYO6 pathway

Increasing evidence indicates that dysregulation of lncRNAs plays a vital role in pathological processes of cancers. Aberrant expression of lncRNAs, such as ANRIL, H19, GAS5, HOTAIR, MALAT1, MEG3, NEAT1, and UCA1, is involved in GC progression [14]. LncRNA UCA1 has gained attention in recent years because of its oncogenic effect in many cancers, including GC [15]. In this study, we explored the potential roles of UCA1 in GC and confirmed that UCA1 regulated GC cell proliferation and apoptosis by targeting MYO6 expression through sponging miR-145 (Fig. 10).

The integrative analysis of GEO and TCGA data shows that UCA1 may be involved in GC progression and may be associated with the prognosis of GC [16]. It has been verified that dysregulated UCA1 promoted the progression of GC through the PI3K/Akt/mTOR signaling pathway or Akt/GSK-3B/cyclin D1 [17, 18]. Sun Li et al. found that lncRNA-UCA1/miR-495-3p/SATB1 regulated GC cell proliferation and invasion [19]. Similarly, UCA1 could accelerate the migration and inhibit cell apoptosis of GC cell lines via the miR-182/TIMP2 pathway [20]. UCA1 also could sponge miR-590-3p to inhibit the growth of GC cells by targeting CREB1 [21]. Moreover, UCA1 could increase chemotherapy resistance to doxorubicin in GC [22]. However, the functional mechanism of UCA1 in GC progression has not been fully elucidated.

Similarly to the above studies, we found that UCA1 was enriched in serum exosomes from GC patients and significantly overexpressed in GC tissues and cell lines. These results are consistent with the analysis based on TCGA and GTEx data. In addition, tumor size, metastasis, and recurrence are associated with UCA1 upregulation in GC patients. Our subsequent investigations indicated that UCA1 overexpression increased GC cell growth, whereas UCA1 knockdown had the opposite effect. The mouse xenograft model also verified these observations of GC growth. Furthermore, downregulation of UCA1 promoted G0/G1 phase arrest and cell apoptosis of GC cells.

LncRNAs can act as miRNAs sponges, influencing miRNAs’ function by suppressing their effect on targeted transcripts [23]. Therefore, we performed bioinformatics analysis to predict miRNAs binding sites on UCA1, and miR-145 was demonstrated to be a potential target of UCA1. Previous studies have revealed that UCA1 regulates cervical cancer cell proliferation, migration, and invasion via targeting miR-145 [24]. Moreover, UCA1 promotes nasopharyngeal carcinoma cell proliferation, invasion, and migration through modulation of miR-145 [25]. In addition, UCA1 promotes migration and invasion of bladder cancer cells via the has-miR-145/ZEB1/2/FSCN1 pathway [26]. However, the effects of the interaction between UCA1 and miR-145 on GC progression have not been studied. Herein, we found that UCA1 and miR-145 were negatively correlated in GC. Luciferase and RIP assays demonstrated that miR-145 could directly bind to UCA1 in GC cells. Moreover, knockdown of UCA1 in GC cells could increase the miR-145 expression. These results revealed that UCA1 could act as a miRNA sponge for miR-145 to inhibit its function in GC cells.

MiRNAs binding to the target mRNAs 3ʹ-UTR could inhibit target gene expression by a post-transcriptional mechanism. miR-145 has been proved to play important roles in GC development through targeting SOX9, SMAD2, KLF5, and CD44 [27–30]. Moreover, Lei Chao et al. found that miR-145 could inhibit GC cell migration and metastasis by suppressing MYO6 [31]. Therefore, we mainly focused on the cell proliferative functions of the miR-145/MYO6 axis in GC. In accordance with previous studies, we observed that miR-145 expression decreased whereas MYO6 expression increased in GC, and MYO6 is a downstream target of miR-145. In addition, we verified that miR-145 inhibited GC cell proliferation and promoted GC cell apoptosis, but MYO6 had the opposite effect. Furthermore, we confirmed that the effect of the miR-145/MYO6 axis on GC cell proliferation and apoptosis was at least partially reversed by UCA1, which has not been reported previously.

This study, however, has potential limitations that could be addressed in the future. Firstly, we did not observe the effect of the UCA1/miR-145/MYO6 axis on GC cell migration and invasion. Secondly, the effect of UCA1 and miR-145 on the prognosis of patients with GC should be studied in the future. Moreover, further studies might be conducted to determine whether the UCA1/miR-145/MYO6 axis plays a role in GC resistance to chemotherapy reagents.

## Conclusions

In summary, this study is the first to demonstrate the regulatory function of the UCA1/miR-145/MYO6 axis in GC. UCA1 affects GC cell proliferation and apoptosis by acting as a sponge of miR-145 to facilitate MYO6 expression. The UCA1/miR-145/MYO6 axis provides insights into the underlying mechanisms of GC progression and may serve as a potential therapeutic target for the treatment of GC development.

## Data Availability

The data supporting the conclusions of this article are available from the corresponding author on reasonable request.

## Abbreviations

CCK-8: Cell Counting Kit-8 assay
GC: Gastric cancer
IHC: Immunohistochemistry
lncRNAs: Long noncoding RNAs
miRNAs: MicroRNAs
miR-145: MicroRNA-145
miR-NC: MiR-145 mimic negative control
MUT: Mutant type
MYO6: Myosin VI
OD: Optical density
Q-PCR: Quantitative real-time PCR
RIP assay: RNA immunoprecipitation assay
SD: Standard deviation
shUCA1: The shRNA oligo targeting UCA1
shNC: The shRNA negative control oligo
si-NC: SiRNA negative control
TCGA: The Cancer Genome Atlas
UCA1: Urothelial carcinoma associated 1
UTR: Untranslated region
WT: Wild type
